# Hypoxia-induced NOS1 as a therapeutic target in hypercholesterolemia-related colorectal cancer

**DOI:** 10.1186/s40170-024-00338-2

**Published:** 2024-05-17

**Authors:** Weiqing Qiu, Li Zhao, Hua Liu, Ping Xu, Changlin Qian

**Affiliations:** 1 Department of General Surgery, Renji Hospital, School of Medicine, Shanghai Jiao Tong University, 2000 Jiangyue Road, Shanghai, 200012 China; 2 Division of Gastroenterology and Hepatology, Key Laboratory of Gastroenterology and Hepatology, Ministry of Health, Renji Hospital, School of Medicine, Shanghai Jiao Tong University, Shanghai Institute of Digestive Disease, Shanghai, 200012 China

**Keywords:** Hypercholesterolemia, Nitric oxide synthase, Hypoxia, Oxidized low-density lipoprotein, Colorectal cancer

## Abstract

**Background:**

It is well established that hypercholesterolemia increases the risk of atherosclerosis, especially because it reduces the availability of nitric oxide (NO). However, the relationship between hypercholesterolemia and NO in regulating colorectal cancer development and progression remains unknown.

**Methods:**

We conducted bioinformatics analysis, qRT-PCR, ChIP-qPCR assays, luciferase report assays, clonogenic survival assays, and multiple mouse models to investigate the function and mechanism of hypercholesterolemia in regulating NO signaling. Additionally, NOS inhibitors were used to evaluate the potential of therapeutic strategy in anti-tumor response.

**Results:**

Here, we show that oxidized low-density lipoprotein (oxLDL) cholesterol and its receptor LOX-1 are essential for hypercholesterolemia-induced colorectal tumorigenesis. Mechanically, the oxLDL promotes the oxidant stress-dependent induction of hypoxia signaling to transcriptionally up-regulate NO synthase (NOS) especially NOS1 expression in colorectal cancer (CRC) cells. More importantly, our results suggested that selective inhibition of NOS1 with its specific inhibitor Nω-Propyl-L-arginine is a suitable therapeutic strategy for hypercholesterolemia-related CRC with both efficacy and toxicity reduction.

**Conclusions:**

Our findings established that hypercholesterolemia induces the oxidant stress-dependent induction of hypoxia signaling to transcriptionally up-regulate NOS1 expression in CRC cells, and the clinically applicable NOS1 inhibitor Nω-Propyl-L-arginine represents an effective therapeutic strategy for hypercholesterolemia-related CRC.

**Supplementary Information:**

The online version contains supplementary material available at 10.1186/s40170-024-00338-2.

## Background

The association between obesity and increased colorectal cancer (CRC) incidence has been evaluated in several epidemiological studies [1]. However, studies have not been able to determine which metabolic disorder increases cancer risk in obese people [2]. A metabolic disorder known as hypercholesterolemia has been linked to an increased CRC risk in obese people [3]. Many studies indicated a positive association between serum total cholesterol levels and the risk of CRC [3–5]. In a case-control study [4] of 129 patients with CRC and 258 matched controls among examinees undergoing a health check-up, there was a significant positive relationship between serum cholesterol levels and the risk of CRC after adjustment for sex, age, BMI, smoking status, and alcohol consumption. It was widely proposed that hypercholesterolemia, oxidative stress, and inflammation are closely related in regulating tumorigenesis [6, 7]. In support of this hypothesis, a previous study reported that hypercholesterolemia promotes the development and progression of CRC by reducing the production of natural killer T cells and *γδ* T cells from hematopoietic stem cells [8]. However, the molecular mechanism underlying hypercholesterolemia-induced colorectal tumorigenesis remains incompletely understood.

Water-soluble nitric oxide (NO) is a ubiquitous gas with free radical groups that plays a variety of physiological roles, such as inflammation and immune responses in the cardiovascular system. In mammalian cells, NO is endogenously produced by using L-arginine as substrate and catalyzed by three types of NO synthase (NOS) encoded by three genes: neuronal NOS (nNOS/NOS-1), inducible NOS (iNOS/NOS-2), and endothelial NOS (eNOS/NOS-3) [9]. Depending on the concentration and time of NO, NO can have both pro- and anticancer effects. A number of tumor-related processes are regulated by NO, including angiogenesis, apoptosis, cell cycle progression, invasion, and metastasis [10]. Previous studies have demonstrated that NO/NOSs signaling plays a crucial role in the development of CRC [11–13]. In particular, aberrant NO/NOSs signaling was frequently detected at inflammatory sites, indicating that NO levels may be higher in inflammatory and tumor tissues [14, 15]. Indeed, it was found that both human colitis and carcinoma tissues expressed higher levels of NOS than non-carcinoma tissues [16–19].

It is well established that hypercholesterolemia increases the risk of atherosclerosis, especially because it reduces the availability of NO [20]. However, the relationship between hypercholesterolemia and NO in regulating cancer development and progression remains unknown. The aim of this study was to further characterize the mechanisms involved in the pro-tumoral effects of hypercholesterolemia-derived NO signaling. We compared the effects of hypercholesterolemia on tissue culture, a mouse xenograft model, and a chemical carcinogenesis mouse model, and identified NOS1 as an unidentified direct target of HIF-1α that contributes to the oncogenic role of hypercholesterolemia in CRC.

## Material and methods

### Mice

Animal experiments were conducted in accordance with the Guide for the Care and Use of Laboratory Animals (National Academies Press, 2011) and were approved by the institutional Animal Care Committee. *ApoE*^−/−^ and wildtype (WT) C57/BL6J mice were fed standard mouse chow (5.4 g fat/100 g diet, 0% cholesterol). High cholesterol diet (HCD)-induced hypercholesterolemic mice were fed a diet with 10 g fat/100 g diet, and 1.25 g cholesterol/100 g.

The azoxymethane (AOM)/dextran sodium sulfate (DSS)-induced CRC experiments were performed as described in previous publications [8]. Three-month-old *ApoE*^−/−^ or WT male C57/BL6J mice were subcutaneously injected with a solution of AOM (Sigma, A5486) at a dose rate of 15 mg/kg body weight, once weekly for 3 successive weeks. 2% of DSS (Yeasen, 60,316) was given in drinking water over 5 days in the last week. Mice were sacrificed 15 weeks after the last injection of AOM. For L-NAME (MedChemExpress, HY-18729) treatment experiments, mice were randomized to be provided with drinking water supplemented with vehicle or 1 g/L of L-NAME at 8 weeks after the last injection of AOM. Based on previous measurements of water consumption and average mouse weight, 1 g/L of L-NAME taken ad libitum corresponds to an approximate dose of 18 mg/kg [21]. Tumor counts and histopathologic staging of tumors were performed by a cancer pathologist in a blind fashion. Blood pressure was measured as the average of five consecutive measurements non-invasively in pre-warmed animals by tail-cuff plethysmography before mice sacrifice.

For Xenograft Models, 1 × 10^6^ HCT116 cells were injected subcutaneously into the 4- to 6-week-old male nude mice. The growth was evaluated weekly, and the tumor volume was measured using the following formula: 0.52 × L × W^2^, where L indicates the length and W indicates the width. For Nω-Propyl-L-arginine (MedChemExpress, HY-102062) treatment experiments, mice were randomized to be provided with drinking water supplemented with vehicle or 1 g/L of Nω-Propyl-L-arginine following tumor size reached 0.15 cm^3^. Tumor volumes and body weights were measured three times weekly to monitor tumor burden and weight loss during treatment. Mice were sacrificed and examined after the last drug administration, and the tumors were harvested for photography, weighing, and immunohistochemistry analysis. Blood pressure was measured as the average of five consecutive measurements non-invasively in pre-warmed animals by tail-cuff plethysmography before mice sacrifice.

### Cell lines

HEK-293 T, HCT116, SW480, and LoVo cell lines were obtained from the Cell Bank in the Chinese Academy of Sciences of China (Shanghai, China) and cultured in RPMI 1640 or Dulbecco’s Modified Eagle’s Medium supplemented with 10% fetal bovine serum, 80 U/ml penicillin, 0.08 mg/ml streptomycin and 2 mM glutamine at 37 °C in a humidified atmosphere containing 5% CO_2_. All cells were regularly tested for Mycoplasma contamination.

### Lentivirus production

The shRNAs were inserted into the pLKO.1-puro vector (Plasmid cat. no. 8453, Addgene Inc., USA) and the empty vector was used as a control. shRNAs sequences are shown in Supplementary Table 1. To produce lentiviruses, HEK-293 T cells were co-transfected with shRNA vector along with the packaging plasmids psPAX2 and VSVG using Lipofectamine 3000 (Invitrogen). After 48 hours and 72 hours, the viral supernatant was harvested and the viral particles were filtered using a 0.45 μm membrane filter. The viral supernatant was stored at − 80 °C.

### Immunohistochemistry

Xenografts and AOM/DSS-induced tumor tissues were removed and fixed with 4% paraformaldehyde for standard histological processing, sectioning, and staining. The primary antibody against Ki-67 (Abcam, ab15580, 1:1000 dilution) was used herein.

### Immunofluorescence

Frozen sections of AOM/DSS-induced tumor tissue were used for immunofluorescence. The slide was first sealed with 10% horse serum for 1 hour and then incubated overnight with HIF-1α antibody (Abcam, ab179483, 1:200) at 4 °C. The sections were incubated with a second antibody (Abcam, ab150075, 1:400) conjugated with appropriate Alexa Fluor 647 at room temperature for 1 hour and then counterstained with DAPI (4′,6′-diamidino-2- phenylindole, Sigma, D9542) and mounted.

### RNA extraction and RT-qPCR

Total RNA was isolated from cells using TRIZOL reagent (Thermo Fisher, 15,596,018) following the manufacturer’s instructions. Reverse transcription was performed using oligo DT and SuperScript™ III reverse transcriptase following the manufacturer’s instructions (Invitrogen, 18,080,051) and quantitative PCR was performed using SYBR Green master mixture (Roche, 4,913,914,001) on LightCycler 480 Instrument. Each sample was tested in triplicate. Primer sequences are listed in Supplementary Table 2.

### Measurement of oxLDL levels

Levels of oxLDL were measured in heparinized blood plasma using a murine oxLDL sandwich enzyme-linked immunosorbent assay (ELISA) kit (Elabscience, E-EL-M0066c) according to the manufacturer’s protocol.

### Proliferation assay

Cell proliferation was determined by Cell Counting Kit-8 (CCK-8, MedChemExpress, HY-K0301). The cells were seeded in 96-well plates with a density of 5 × 10^3^ cells per well and cells were incubated with different concentrations of L-NAME (MedChemExpress, HY-18729) or Nω-Propyl-L-arginine (MedChemExpress, HY-102062) for 48 hours. CCK-8 was added at a fixed ratio of 1:10 to each well of the 96-well plate, followed by further incubation for 2 hours. The absorbance was then measured in a microplate reader (Thermo Fisher Scientific, Inc.) at 450 nm.

### Clonogenic survival assay

HCT116 cells were seeded in 6-cm dishes at a density of 600 cells per well. Cells were then incubated with different concentrations of L-NAME (MedChemExpress, HY-18729) or Nω-Propyl-L-arginine (MedChemExpress, HY-102062) for 7 days and stained with 0.5% crystal violet.

### Chromatin immunoprecipitation (ChIP)

All ChIP experiments were performed as previously described [22]. Briefly, HCT116 cells were cross-linked with 1% formaldehyde for 15 minutes at room temperature and then quenched with glycine with a final concentration of 125 mM for 10 minutes at room temperature followed by cell lysis and sonication. Immunoprecipitation was performed using anti-HIF-1α antibody (Cell Signaling Technology, 36,169) or rabbit IgG (Santa Cruz Biotechnology, sc-2027) for negative control. DNA was recovered using a PCR purification kit (Qiagen, 28,106). Input samples were treated in the same way except that no immunoprecipitation was performed.

### Luciferase reporter assay

For HRE luciferase reporter assay, the 1000 bp upstream of the transcriptional start site (TSS) of the human NOS1 gene was cloned into the pGL4.10-basic vector (NOS1-HRE-WT) using the method described previously [23]. Successive deletion of HRE was made in the NOS1-HRE-WT construct using PCR-directed mutagenesis (NOS1-HRE-Del). HCT116 cells were transiently transfected with Firefly luciferase plasmid: pGL4.10-basic vector, NOS1-HRE-WT plasmid, or the ΔHRE truncation, as well as phRL-PBGD Renilla luciferase plasmid for internal control. After 24 hours, the cells were incubated with or without oxLDL (Yeasen, 20605ES05) for 12 hours. Luciferase activity was measured using the Dual-Luciferase Assay Kit (Promega, E1910) according to the manufacturer’s protocol.

### Gene expression analysis

TCGA RNA-seq expression and clinical data were downloaded either from cBioPortal for Cancer Genomics (http://cbioportal.org). The gene expression profiles of GSE96528 and GSE75315 and the ChIP-sequencing profiles of GSE144189 were obtained from the Gene Expression Omnibus (GEO, http://www.ncbi.nlm.nih.gov/geo/). The hypoxia signature was computed as the average expression (z-score) of 34 hypoxia-inducible genes [24] represented by ADM, ALDOC, BNIP3, BNIP3L, CA9, CA12, CP, COL5A1, DDIT4, EGLN3, ENO2, GYS1, HK2, HMOX1, LOX, LDHA, LRP1, MUC1, MXI1, NDU-FA4L2, NDRG1, NRN1, PFKFB4, PPP1R3C, POU5F1, PDK1, RAB20, SLC7A5, SLC2A1, SLC16A3, STC2, VEGFA, TXNIP, ZNF395.

### Statistical analysis

All statistical analyses and data visualization were performed using GraphPad Prism 9.0 software. The Student’s t-test or one-way analysis of variance (ANOVA) multiple comparisons were used for the comparison of different groups. The Chi-squared (*χ2*) test was used for the comparison of proportions and the Pearson’s correlation was used to compare the expression of genes. The Log-rank test was used for the comparison of survival curves. Data were expressed as means ±SD. The difference was statistically significant at the 0.05 level (*p* < 0.05).

## Results

### Hypercholesterolemia increases the incidence and histopathologic severity of colorectal neoplasia

**Fig. 1:**
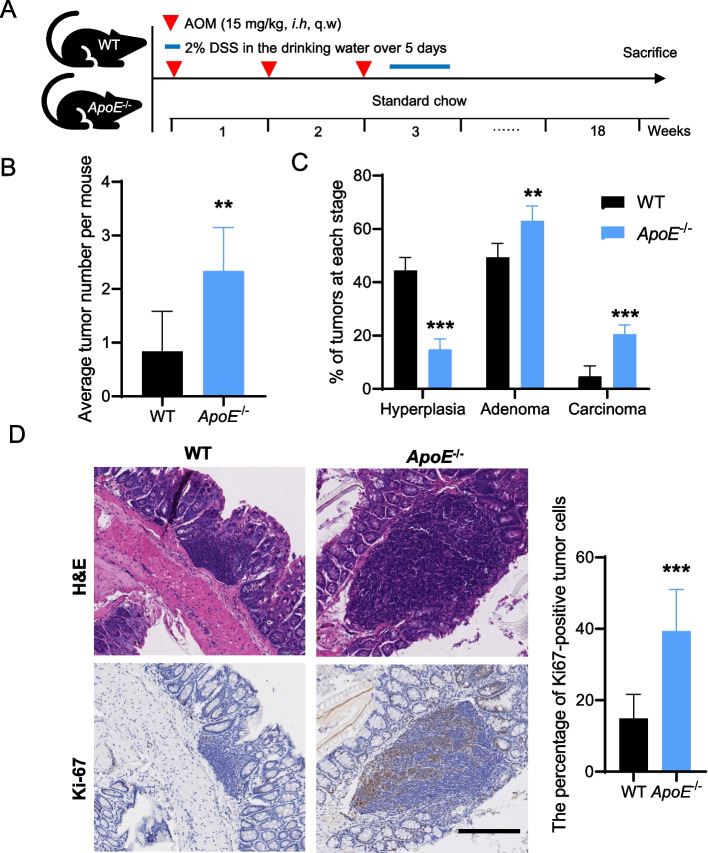
Hypercholesterolemia increases the incidence and histopathologic severity of colorectal neoplasia. **A** Study design of the used hypercholesterolemic ApoE^−/−^ murine model. *N* = 6. **B** Average tumor number per mouse from WT and ApoE^−/−^ mice. **C** Histopathologic stages of the tumors from WT and ApoE^−/−^ mice. **D** Representative images of H&E and Ki-67 immunohistochemical staining for CRC samples from WT and ApoE^−/−^ mice. Scale bars, 200 μm. Two-tailed Student’s t-test (**B** and **C**). Error bars represent ± SD. **p < 0.01, ***p < 0.001. Data represent mean ± SD of at least three independent experiments

To determine the potential role of hypercholesterolemia in CRC, we first induced colorectal tumorigenesis with AOM/DSS in a well-established mouse model of hypercholesterolemia, the *ApoE*^−/−^ mouse (Fig. 1A and Supplementary Fig. S1). Mice with hypercholesterolemia developed almost two times more tumors than wild-type (WT) mice (Fig. 1B), suggesting that hypercholesterolemia appears to promote colorectal tumorigenesis. Pathological results showed that the tumors at the late stage of tumorigenesis, carcinoma, constituted less than 10% in WT mice. However, more than 20% carcinoma was found in *ApoE*^−/−^ mice, indicating that the carcinogenesis rate was significantly higher in the hypercholesterolemic mice than in WT mice (Fig. 1C). The expressions of Ki-67 in carcinoma tissues from both hypercholesterolemic and WT mice was detected by immunohistochemical staining. The results showed that Ki-67 expression in hypercholesterolemic mice was significantly higher than that in WT mice, indicating that hypercholesterolemia could promote CRC cell proliferation (Fig. 1D). Generally, hypercholesterolemia significantly promotes the occurrence and progression of CRC.

### oxLDL/LOX-1 links hypercholesterolemia and CRC aggressiveness

**Fig. 2:**
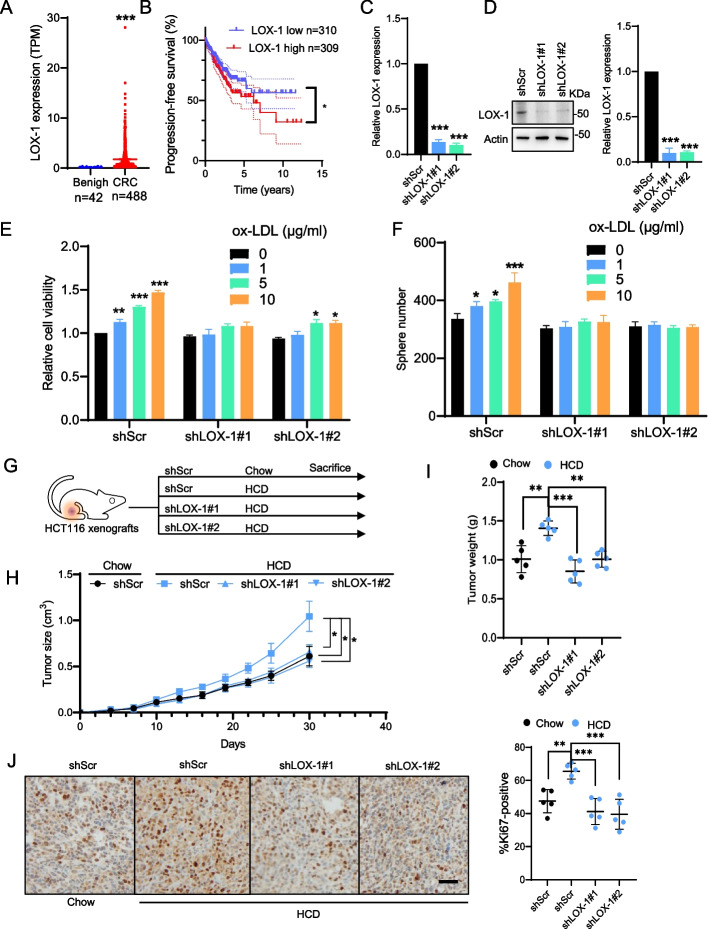
oxLDL/LOX-1 links hypercholesterolemia and CRC aggressiveness. **A** Dot plots for the mRNA expression levels of LOX-1 according to normal vs. tumor. The mRNA expression values were obtained from the TCGA-CRC dataset. **B** Progression-free survival according to LOX-1 mRNA expression for CRC patients from the TCGA-CRC database. **C** RT-qPCR analysis of LOX-1 mRNA expression in LOX-1 knockdown and parental HCT116 cells. **D** Western blot analysis of LOX-1 protein expression in LOX-1 knockdown and parental HCT116 cells. **E** The effect of oxLDL treatment on cell viability in LOX-1 knockdown and parental HCT116 cells. **F** The effect of oxLDL treatment on clonogenic survival in LOX-1 knockdown and parental HCT116 cells. **G** Study design of the used HCD-induced hypercholesterolemia murine model. **H** Tumor growth of shScr and shLOX-1 cells xenografted in HCD-induced hypercholesterolemia nude mice. **I** Tumor weight of shScr and shLOX-1 cells xenografted in HCD-induced hypercholesterolemia nude mice. **J** Representative images of immunohistochemistry for Ki67 staining in xenografts. Scale bars, 200 μm. Quantification of positive signals is provided on the right of the representative images. Two-tailed Student’s t-test (**A, C, E, F, I and J**), Log-rank test (**B**), One-way ANOVA (H). Error bars represent ± SD. *p < 0.05, **p < 0.01, ***p < 0.001. Data represent mean ± SD of at least three independent experiments

Hypercholesterolemia is characterized by increased serum low-density lipoprotein cholesterol (LDL), which is susceptible to oxidation by reactive oxygen species (ROS) [25]. It has been discovered that oxidized LDL (oxLDL) is a prominent pathogenic factor in cardiovascular diseases as well as various types of cancer [26]. According to a previous study, increased oxLDL levels promote the development of cancer through binding to its receptor LOX-1 and activating cellular pathways involved in inflammation, proliferation, and invasion/migration [27]. Thus, we speculate that the oxLDL/LOX-1 axis may mediate the function of hypercholesterolemia to promote colorectal tumorigenesis. We first analyzed the mRNA expression of LOX-1 in the TCGA-CRC cohort, and found that LOX-1 was significantly elevated in CRC tissues (Fig. 2A). Additionally, Kaplan-Meier analysis of progression-free survival (PFS) revealed that high expression of LOX-1 was associated with shorter progression-free survival in CRC patients (*P* < 0.05; Fig. 2B). To determine the functional importance of oxLDL/LOX-1 in colorectal tumorigenesis, LOX-1 was knocked down using small-hairpin RNAs (shRNA) (Fig. 2C and D) in HCT116 cells and the cell growth of the resulting cells was assessed. As depicted in Fig. 2E and F, oxLDL treatment indeed promoted cell growth and aggressiveness, as evidenced by CCK-8 and clonogenic survival assays. In addition, LOX-1 ablation significantly compromised oxLDL-mediated cell growth and survival. Further confirmation of the in vitro results was provided by the HCT116 CRC xenograft nude mouse model with high-cholesterol diet (HCD)-induced hypercholesterolemia (Fig. 2G and Supplementary Fig. S2A). As reflected by the tumor growth curve and Ki-67 immunohistochemical staining analysis, LOX-1 deletion remarkably compromised the proliferation of CRC induced by hypercholesterolemia (Fig. 2H-J and Supplementary Fig. S2B).

### NO/NOS signaling is upregulated by oxLDL treatment and is needed for hypercholesterolemia-induced colorectal tumorigenesis

**Fig. 3:**
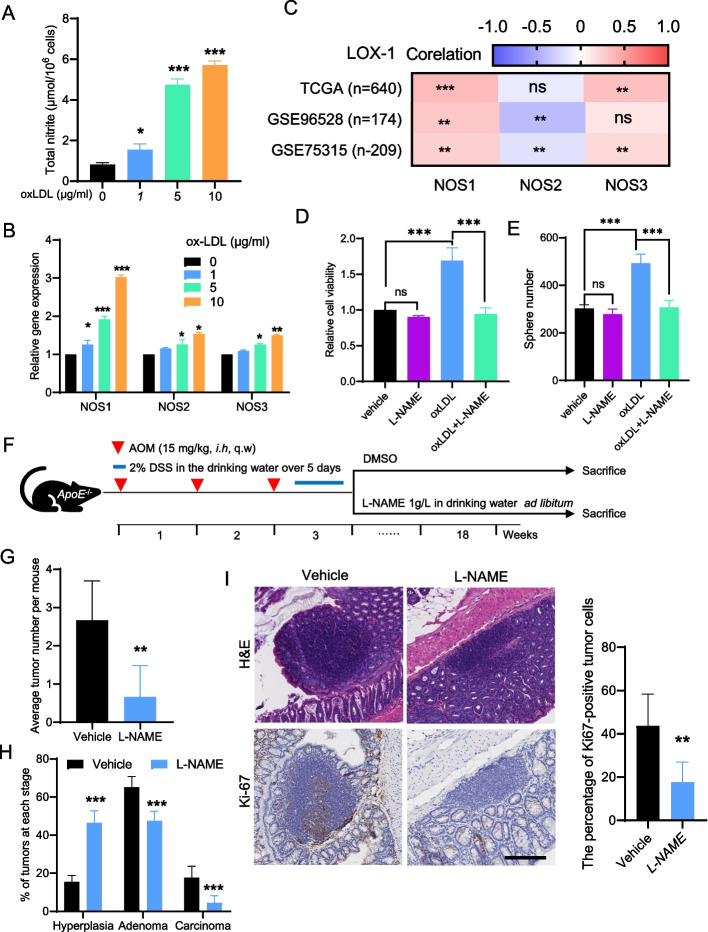
NO/NOS signaling is upregulated by oxLDL treatment and is needed for hypercholesterolemia-induced colorectal tumorigenesis**. A** Changes of total nitrite levels in oxLDL-treated HCT116 cells. **B** RT-qPCR analysis of NOS mRNA expression in oxLDL-treated HCT116 cells. **C** Correlation between LOX-1 and NOS mRNA expression in CRC samples from different CRC datasets. **D** The effect of oxLDL with or without L-NAME treatment on cell viability in HCT116 cells. **E** The effect of oxLDL with or without L-NAME treatment on clonogenic survival in HCT116 cells. **F** Study design of the used hypercholesterolemic ApoE^−/−^ mice treated with vehicle or L-NAME. **G** Average tumor number per mouse from vehicle- or L-NAME-treated ApoE^−/−^ mice. **H** Histopathologic stages of the tumors from vehicle- or L-NAME-treated ApoE^−/−^ mice. **I** Representative images of H&E and Ki-67 immunohistochemical staining for CRC samples from vehicle- or L-NAME-treated ApoE^−/−^ mice. Scale bars, 200 μm. Two-tailed Student’s t-test (A, B, D, E, G, and H), Pearson’s correlation (B). Error bars represent ± SD. ns = not significant, *p < 0.05, **p < 0.01, ***p < 0.001. Data represent mean ± SD of at least three independent experiments

The activation of NO/NOS signaling under several stress conditions contributes to the development of atherosclerosis. The overproduction of oxLDL led to imbalanced activation of NO/NOS signaling, which represents the major factor triggering the causative processes of atherosclerosis accompanying vascular endothelial inflammation and dysfunction [28–30]. To investigate whether oxLDL has a function in regulating NO signaling in CRC cells, HCT116 cells were incubated with 0, 1, 5, or 10 μg/ml oxLDL for 24 hours. As indicated in Fig. 3A, oxLDL treatment significantly stimulated NO production in HCT116 cells in a dose-dependent manner. However, this effect was overcome by using the non-selective NOS inhibitor L-NAME (Supplementary Fig. S3). NOSs, especially NOS1, expression was also markedly up-regulated by oxLDL treatment (Fig. 3B), which is consistent with the analysis based on the TCGA-CRC database (Fig. 3C). Previous studies indicated that the development and progression of CRC are accompanied by metabolic disturbances in the L-arginine/NO pathway, potentially applicable as prognostic biomarkers and therapeutic targets [31–33]. We then asked whether NO/NOS signaling is needed for hypercholesterolemia-induced colorectal tumorigenesis. To this end, HCT116 cells were incubated with oxLDL for 24 hours with or without L-NAME, and the enzymatic activity of NOS, cellular viability, and aggressiveness were examined. As indicated in Fig. 3D and Fig. 3E, L-NAME significantly suppressed the increased cellular viability and aggressiveness of HCT116 cells caused by oxLDL treatment. In order to verify the results of the experiment in vivo, we employed the AOM/DSS-treated *ApoE*^−/−^ mouse model to further confirm the findings in vitro (Fig. 3F). As demonstrated in Fig. 3G and Fig. 3H, L-NAME treatment significantly compromised the incidence and progression of CRC induced by hypercholesterolemia. This effect was further confirmed by HE and Ki-67 immunohistochemical staining in the mouse CRC samples (Fig. 3I). There were no significant differences in body weight between treatment groups (Supplementary Fig. S4A); however, the blood pressure was greatly increased when mice were treated with L-NAME (Supplementary Fig. S4B), which is consistent with the observation that L-NAME was used to establish a mouse model of hypertension [34]. Thus, pan-NOS inhibition by L-NAME reduces tumor burden but increases blood pressure in an AOM/DSS-induced hypercholesterolemic CRC mouse model.

### Hypercholesterolemia activates hypoxia signaling to upregulate NOSs expression

**Fig. 4:**
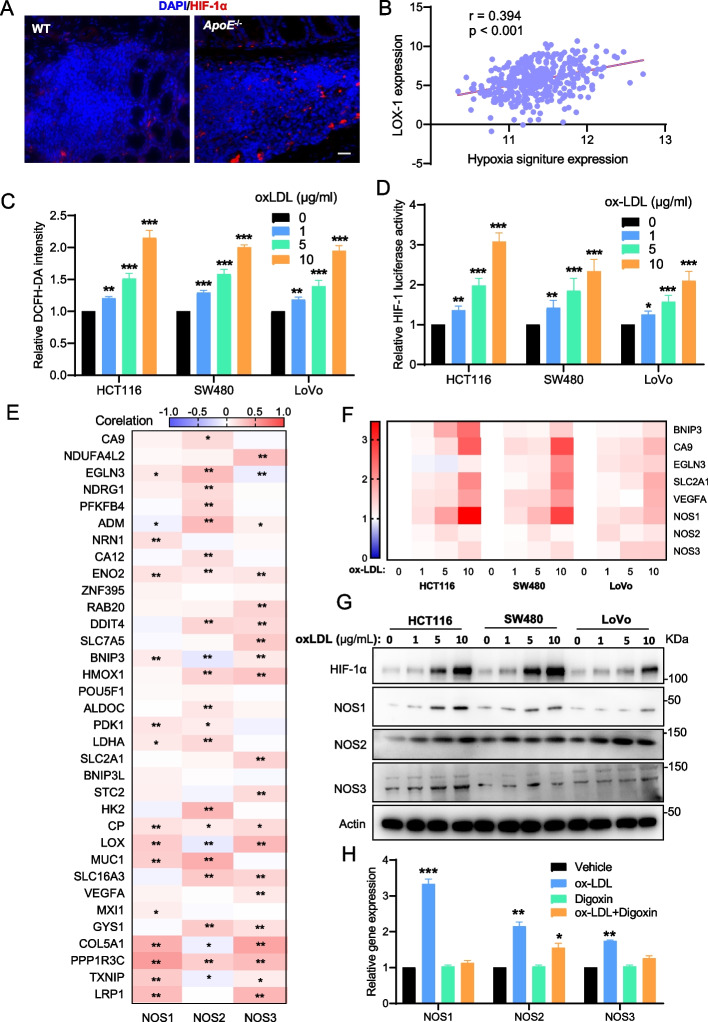
Hypercholesterolemia activates hypoxia signaling to upregulate NOS expression. **A** Representative immunofluorescence images of HIF-1α staining in CRC samples from WT and ApoE^−/−^ mice. **B** Correlation between LOX-1 mRNA expression and the hypoxia signature in CRC patients from the TCGA-CRC dataset. **C** Intracellular levels of ROS were measured by DCFH-DA staining in indicated cells treated with oxLDL. **D** HIF-1 pathway activity was measured by HIF-1 luciferase reporter assay in indicated cells treated with oxLDL. **E** Correlation between mRNA expression of NOSs and HIF target genes in CRC samples from TCGA-CRC datasets. **F** RT-qPCR analysis of the mRNA expression of NOSs and HIF target genes in indicated cells treated with oxLDL. **G** Western blot analysis of the protein expression of NOSs and HIF-1α in indicated cells treated with oxLDL for 24 h. **H** RT-qPCR analysis of the mRNA expression of NOSs in indicated oxLDL-treated cells incubated with or without digoxin. Pearson’s correlation (B and E), Two-tailed Student’s t-test (C, D, and H). Error bars represent ± SD. *p < 0.05, **p < 0.01, ***p < 0.001. Data represent mean ± SD of at least three independent experiments

Next, we investigated how hypercholesterolemia triggers NO/NOS dysregulation in CRC. Hypoxia, as an important pathophysiological condition, is closely linked to hyperlipidemia and atherosclerotic development. It is still not known whether hypoxia signaling is also involved in this process. Interestingly, NOSs especially NOS2 and NOS3 are relevant for vascular NO production in certain settings under hypoxic conditions [35]. According to the classical view, NO is thought to increase local blood flow and oxygen delivery by relaxing vascular smooth muscle. To examine whether hypoxia is relevant to hypercholesterolemia-induced NO/NOS dysregulation in CRC, we investigated CRC specimens from the AOM/DSS-treated *ApoE*^−/−^ hypercholesterolemic mouse model. Indeed, by staining anti-HIF-1α immunofluorescence in the AOM-induced CRC model, we observed that CRC tumors in *ApoE*^−/−^ mice display higher HIF1-α expression than those in WT mice (Fig. 4A). Indeed, we also found a close correlation between the hypoxia signature score and the expression of LOX-1 in human CRC specimens supporting our assumption (Fig. 4B). These results demonstrated that hypercholesterolemia was significantly correlated with the activation of hypoxia signaling in CRC tissues. Next, we sought to explore the downstream regulatory mechanisms underlying the role of hypoxia in regulating NO/NOS expression. Important new insights demonstrate that ROS can stabilize HIF-1α under both hypoxic as well as normoxic conditions. For example, it has been shown in macrophages that oxLDL can trigger HIF-1α accumulation even under normoxic conditions through redox mechanisms [36]. Given that chemotherapy-mediated intracellular upregulation of ROS activates hypoxia signaling and then confers cancer cells to chemotherapy resistance [37, 38], we speculated that hypercholesterolemia may also upregulate intracellular ROS levels to activate the hypoxia signaling and subsequent NO/NOS expression in CRC. To this end, we then quantified the intracellular ROS level and HIF-1 transcriptional activity of CRC cells upon oxLDL treatment through DCFH-DA staining and luciferase reporter assays, respectively. In agreement, oxLDL treatment increased cellular ROS levels and HIF-1 transcriptional activity in HCT116, SW480, and LoVo cells (Fig. 4C and Fig. 4D). Because CRC is known to have hypoxic environments [39], we then investigated the role of HIF signaling in regulating hypercholesterolemia-induced NOS expression. In line with the hypothesis that hypercholesterolemia activates HIF signaling, we found a strong correlation between NOSs and 34 known HIF target genes expression in human CRC (Fig. 4E). Using a panel of CRC cell lines, we detected significant upregulation of NOS and HIF signaling expression under oxLDL treatment conditions in a concentration-dependent manner (Fig. 4F, Fig. 4G, and Supplementary Fig. S5A-C). To confirm the role of the HIF-1 pathway in hypercholesterolemia-induced NOS expression, we used digoxin to inhibit the translation of HIF-1α [40]. Figure 4H and Supplementary Fig. S5D demonstrate that treatment with digoxin reduced the \ NOS expression and NO production in HCT116 cells exposed to oxLDL, alluding to a causal link between HIF signaling and hypercholesterolemia-induced NOS expression. Altogether, these data highlight that hypoxia signaling is a pivotal driver of NO/NOS activation in hypercholesterolemia-related CRC cells.

### NOS1 is a direct target of HIF-1α

**Fig. 5:**
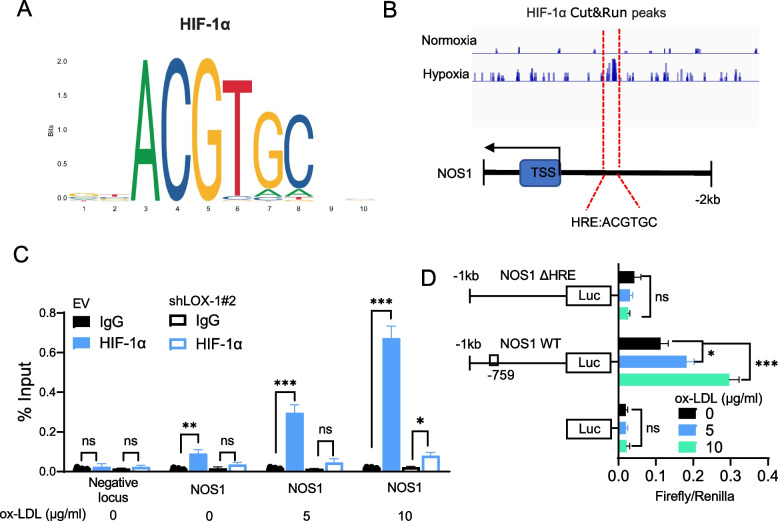
NOS1 is a direct target of HIF-1α. **A** The consensus DNA-binding motif of HIF-1α was obtained from the JASPAR database (http://jaspar.genereg.net/). **B** HIF-1α Cut&Run sequencing peaks on the NOS1 gene in Hep3B cells. The putative consensus HIF-1α binding motif in the NOS1 promoter region (− 759 bp upstream of the TSS). Data were derived from the HIF-1α Cut&Run-sequencing dataset generated from Hep3B cells and downloaded from the Gene Expression Omnibus (GEO, GSE228175). **C** ChIP-qPCR assays on the NOS1 promoter were performed and quantified by qPCR in LOX-1 knockdown and parental HCT116 cells treated with or without oxLDL for 24 hours. **D** Relative luciferase activities of indicated NOS1 promoter reporters in HCT116 cells treated with or without oxLDL for 24 hours. Two-tailed Student’s t-test (C and D). Error bars represent ± SD. *p < 0.05, **p < 0.01, ***p < 0.001. Data represent mean ± SD of at least three independent experiments

Previous studies have demonstrated that NOS2 and NOS3 are transcriptionally regulated by HIF-1α in ischemic heart conditions [35]. Given our above observations that NOS1 was the major upregulated NOS under hypercholesterolemic conditions, it is necessary to explore whether NOS1 is a direct target of HIF-1α. To understand how HIF-1α may regulate NOS1 expression, we analyzed the first 1 kb region upstream of the transcriptional start site (TSS) of the NOS1 gene, in which functional transcription binding sites are most likely to be enriched. Interestingly, the NOS1 promoter contains a canonical HIF-1α binding site called hypoxia response element (HRE), as predicted by the JASPAR database of transcription factor binding profile (Fig. 5A) and Cut&Run sequencing dataset (GSE228175) generated from Hep3B cells (Fig. 5B). Next, ChIP assays with antibodies against HIF-1α were analyzed by q-PCR using primers spanning the HRE within the NOS1 promoter in HCT116 cells. The ChIP-qPCR findings confirmed the direct binding of HIF-1α to the NOS1 promoter in vitro and oxLDL treatment effectively increased HIF-1α binding to the NOS1 gene, while knockdown of LOX-1 effectively abolished this effect (Fig. 5C). Furthermore, the luciferase reporter assay showed that oxLDL treatment facilitated the transcription of NOS1 mediated by HIF-1α. As depicted in Fig. 5D, oxLDL treatment caused a significant increase in the luciferase activity of a WT NOS1 reporter, but no marked change in luciferase activity was observed when the HRE was mutated. Collectively, these data indicated that HIF-1α directly binds to the NOS1 promoter and facilitates NOS1 transcription under hypercholesterolemic conditions.

### Pharmacologic blockade of NOS1 is a promising therapeutic strategy for hypercholesterolemia-related CRC

**Fig. 6:**
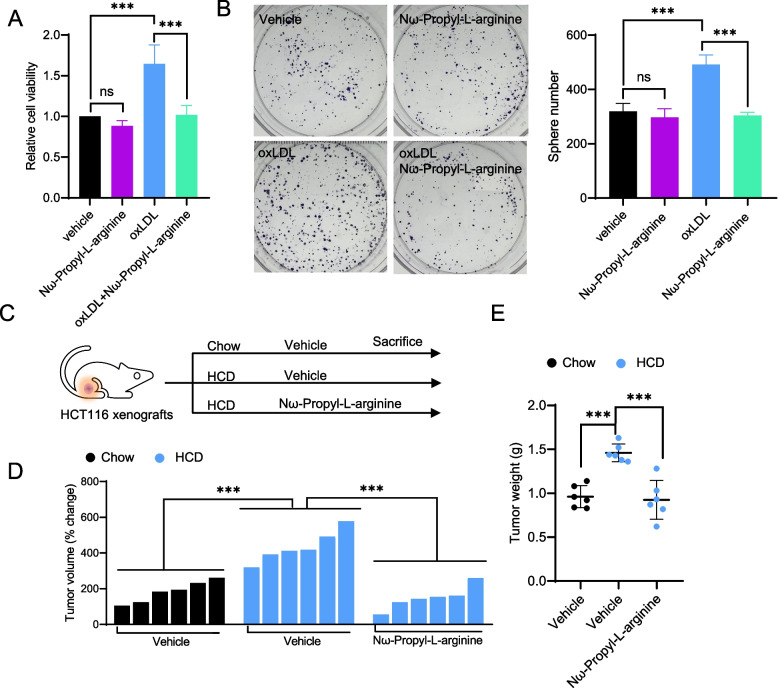
Pharmacologic blockade of NOS1 is a promising therapeutic strategy for hypercholesterolemia-related CRC. **A** Cell growth and **B** colony formation of oxLDL-treated HCT116 cells incubated with or without Nω-Propyl-L-arginine. **C** Study design of the used HCD-induced hypercholesterolemia murine model. **D** Waterfall plot of the response of HCT116 xenografts at 4 weeks after the starting dose of Nω-Propyl-L-arginine administration. **E** Tumor weight of vehicle- or Nω-Propyl-L-arginine-treated mice at 4 weeks after the starting dose of drug administration. Two-tailed Student’s t-test (A, B, D, and E). Error bars represent ± SD. **p < 0.01, ***p < 0.001. Data represent mean ± SD of at least three independent experiments

Of note, our study found that NOS1 is the most significantly up-regulated NOS in hypercholesterolemia-related CRC models, which means that NOS1 may act as an important regulator in the development of hypercholesterolemia-related CRC. In addition, nonselective inhibition of NOS activity might generate severe side effects, which may limit the scope of applications of pan-NOS inhibitors in hypercholesterolemia-related CRC [41]. Therefore, we postulate that the selective inhibition of NOS1 may be a suitable therapeutic strategy for hypercholesterolemia-related CRC with both efficacy and toxicity reduction. To this end, we employed the highly selective NOS1 inhibitor Nω-Propyl-L-arginine to determine its potential therapeutic effect on hypercholesterolemia-related CRC. First, our results found that the cancer-promoting effect of oxLDL on HCT116 cells was significantly inhibited by Nω-Propyl-L-arginine administration (Fig. 6A and Fig. 6B), as evidenced by CCK-8 and clonogenic survival assays. To validate the in vivo anti-cancer activity of Nω-Propyl-L-arginine, the HCT116 CRC xenograft nude mouse model with HCD-induced hypercholesterolemia was employed (Fig. 6C and Supplementary Fig. S6A). In agreement with the in vitro data, Nω-Propyl-L-arginine administration was capable of retarding tumor growth and decreasing tumor burden in mice fed with HCD (Fig. 6D, Fig. 6E, and Supplementary Fig. S6B). Moreover, no apparent toxicity of Nω-Propyl-L-arginine treatment was observed at a dosage of 18 mg/kg, considering that no significant body weight or blood pressure change was observed (Supplementary Fig. S6C and Supplementary Fig. S6D). Collectively, the pharmaceutical blockade of NOS1 through its specific inhibitor Nω-Propyl-L-arginine is a promising therapeutic strategy for hypercholesterolemia-related CRC.

## Discussion

**Fig. 7:**
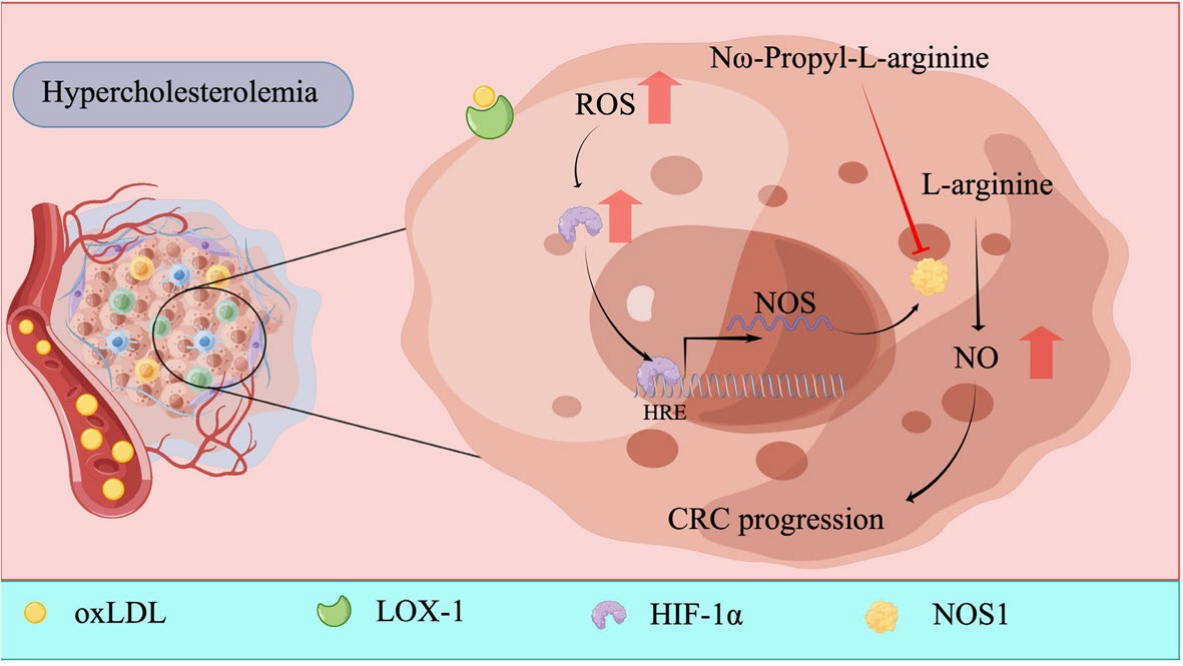
Schematic diagram delineating hypercholesterolemia induces the oxidant stress-dependent induction of hypoxia signaling to transcriptionally up-regulate NOS1 expression in CRC cells, and the selective NOS1 inhibitor Nω-Propyl-L-arginine represents an effective therapeutic strategy for hypercholesterolemia-related CRC

The relationship between the incidence of cardiovascular diseases and cancer is under active investigation and is referred to as cardiooncology [42]. Hypercholesterolemia as a common metabolic abnormality of cardiovascular disease has been shown to increase the risk of cancer-related mortality [43]. Herein, we demonstrate that hypercholesterolemia promotes CRC tumorigenesis by inducing oxidant stress-dependent induction of hypoxia signaling in CRC cells (Fig. 7). This induction of hypoxia signaling transcriptionally upregulates NOSs, especially NOS1, which is critical to hypercholesterolemia-induced CRC growth and survival. Our findings reveal a novel mechanism by which NO/NOS signaling can be regulated by a common metabolic abnormality in CRC and provide insights into new therapeutic strategies for this disease.

Hypoxia is one of the most important characteristics of solid tumors and contributes to cancer initiation, progression, and treatment resistance [44–47]. Unlike NOS1, multiple studies have reported that the expression of NOS2 and NOS3 can be modulated by hypoxia, oxidative stress, and inflammatory cytokines in the cardiovascular system [35, 48]. The relationship between hypoxia signaling and NOS1 expression is poorly investigated. In accordance with our findings, a recent study revealed that hypoxia induces NOS1 expression in astrocytes of the human corpus callosum [49], but the underlying molecular mechanisms have not been elucidated. In this study, our results showed that although all three NOSs exist and contribute to total NO production in CRC cells, only NOS1 was shown to be the main mediator of hypercholesterolemia-induced CRC. Through a series of experiments, we demonstrated that HIF-1α binds to the NOS1 promoter and facilitates NOS1 transcription, providing direct evidence that hypoxia signaling regulates NOS1 expression via HIF-1α in CRC cells.

The pharmacologic blockade of NOS function for cancer therapy has been widely tested in preclinical and clinical settings [21, 50, 51]; however, our study suggests that close attention should be given when targeting all NOS isoforms as a therapeutic intervention for CRC. Our findings further confirmed that the selective blockade of NOS1 is sufficient for inhibiting tumor progression in hypercholesterolemia-related CRC. Increasing evidence suggests that NOS1 plays a critical pro-oncogenic role in multiple types of cancer, but little has been related to CRC [52–54]. A recent study demonstrated that NOS1 is involved in chemotherapy resistance in CRC cells and that NOS1-induced apoptotic resistance can be overcome by NOS1-specific inhibitors [11]. In addition to promoting tumorigenesis by synthesizing a low level of NO, NOS1 can activate intracellular signaling pathways important in regulating cell proliferation and survival by selective S-nitrosylation of key regulatory proteins [52, 55, 56]. S-nitrosylation is an important protein post-translational modification that results from the covalent attachment of an NO group to the thiol side chain of a cysteine residue [57], which is shown to be the most widespread and functionally important form of NO-mediated cellular signals [58]. For example, tumor suppressor PTEN could be selectively S-nitrosylated by NOS1 at Cys83 under low levels of NO conditions [55, 56]. Consequently, S-nitrosylation of PTEN leads to its rapid degradation through the ubiquitin-proteasome system, which contributes to the hyper-activation of the PI3K/Akt cascade in cancer cells [56]. Furthermore, Gao and colleagues reported that the selective S-nitrosylation of phosphofructokinase at Cys351 by NOS1 stabilized the tetramer of phosphofructokinase and resulted in the metabolic rewiring of ovarian cancer [52], highlighting a critical and distinct role of NOS1 in promoting tumor progression. One possible reason for the special ability of NOS1 to S-nitrosylate substrates could be attributed to the unique PDZ domain possessed by NOS1 [55]. The PDZ domain facilitates selective and effective interactions between NOS1 and its target proteins, thereby selectively modulating special cell signaling pathways and biological processes. Thus, protein S-nitrosylation by NOS1 may be critical for hypercholesterolemia-induced tumor promotion in CRC.

## Conclusions

Collectively, our results established that hypercholesterolemia induces the oxidant stress-dependent induction of hypoxia signaling to transcriptionally up-regulate NOS1 expression in CRC cells, and the clinically applicable NOS1 inhibitor Nω-Propyl-L-arginine represents an effective therapeutic strategy for hypercholesterolemia-related CRC.

### Supplementary Information

**Supplementary material 1.**

## Data Availability

All data needed to evaluate the conclusions in the paper are present in the paper and/or the Supplementary Materials. Additional data related to this paper may be requested from the corresponding authors.

## Abbreviations

CRC: Colorectal cancer
NO: Nitric oxide
NOS: Nitric oxide synthesas
oxLDL: Oxidized low-density lipoprotein
AOM: Azoxymethane
DSS: Dextran sodium sulfate
ROS: Reactive oxygen species
HCD: High-cholesterol diet
TSS: Transcriptional start site
HRE: Hypoxia response element
ChIP: Chromatin immunoprecipitation
WT: Wild type
