# Supplementary material for: Hypoxia-induced NOS1 as a therapeutic target in hypercholesterolemia-related colorectal cancer
Source: Cancer Metab. 2024 May 17;12:14. doi: 10.1186/s40170-024-00338-2 (PMC11100240; doi:10.1186/s40170-024-00338-2)
Supplement: Supplementary file 1 — Supplementary material 1. [file 40170_2024_338_MOESM1_ESM.docx]

**Supporting Information**

**Hypoxia-induced NOS1 as a therapeutic target in hypercholesterolemia-related colorectal cancer**

Weiqing Qiu, Li Zhao, Hua Liu, Ping Xu, Changlin Qian

**This file includes:**

Supplementary Figures S1 to S6

Supplementary Tables S1 to S2

**Supplementary Fig. S1**

**
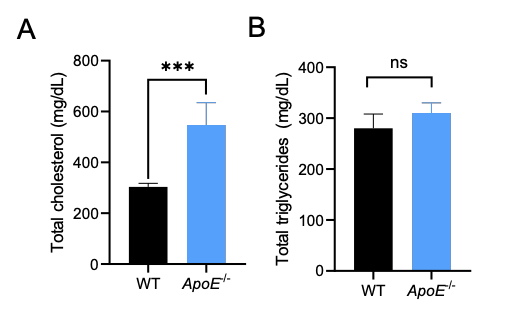
**

**Supplementary Fig. S1.** The serum total cholesterol **(A)** and triglycerides **(B)** levels in WT and ApoE^−/−^ mice. Two-tailed Student’s t-test. Error bars represent ± SD. ****p* < 0.001, ns: not significant. Data represent mean ± SD of at least three independent experiments.

**Supplementary Fig. S2**

**
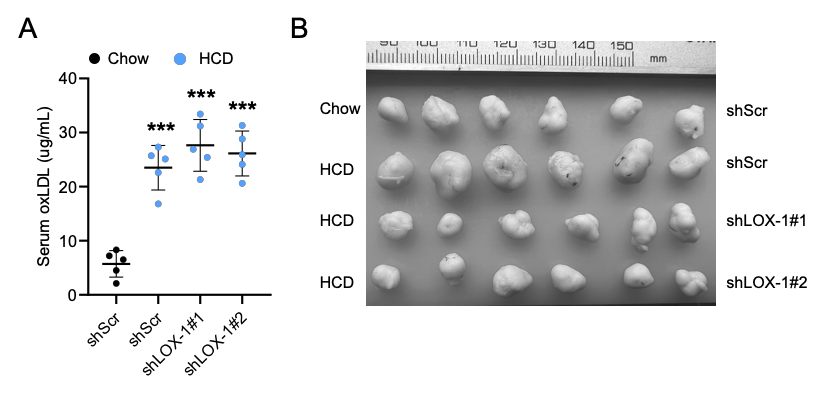
**

**Supplementary Fig. S2. oxLDL/LOX-1 links hypercholesterolemia and CRC aggressiveness. (A)** The serum oxLDL levels in HCD-induced hypercholesterolemia nude mice **(B)** Representative images of xenograft tumors of shLOX-1 knockdown and parental HCT116 cells formed in HCD-induced hypercholesterolemia nude mice (n = 6 mice per group). Two-tailed Student’s t-test. Error bars represent ± SD. ****p* < 0.001. Data represent mean ± SD of at least three independent experiments.

**Supplementary Fig. S3**

**
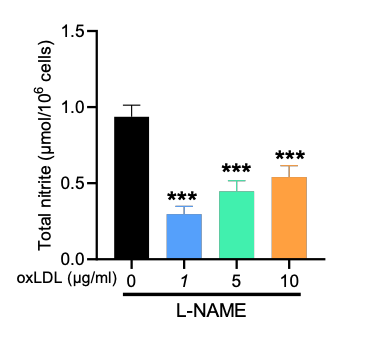
**

**Supplementary Fig. S4.** The non-selective NOS inhibitor L-NAME significantly decreased NO production stimulated by oxLDL treatment. Two-tailed Student’s t-test. Error bars represent ± SD. ns: not significant, ***p < 0.001. Data represent mean ± SD of at least three independent experiments.

**Supplementary Fig. S4**

**
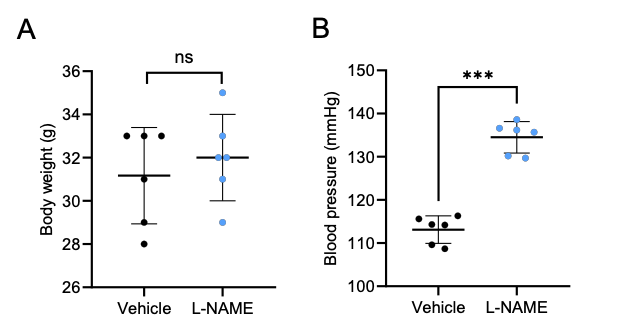
**

**Supplementary Fig. S4. L-NAME treatment causes hypertension in mice. (A)** Body weight of vehicle- or L-NAME-treated mice at 4 weeks after the starting dose of drug administration. **(B)** Blood pressure of vehicle- or L-NAME-treated mice at 4 weeks after the starting dose of drug administration. Two-tailed Student’s t-test. Error bars represent ± SD. ns: not significant, ***p < 0.001. Data represent mean ± SD of at least three independent experiments.

**Supplementary Fig. S5**

**
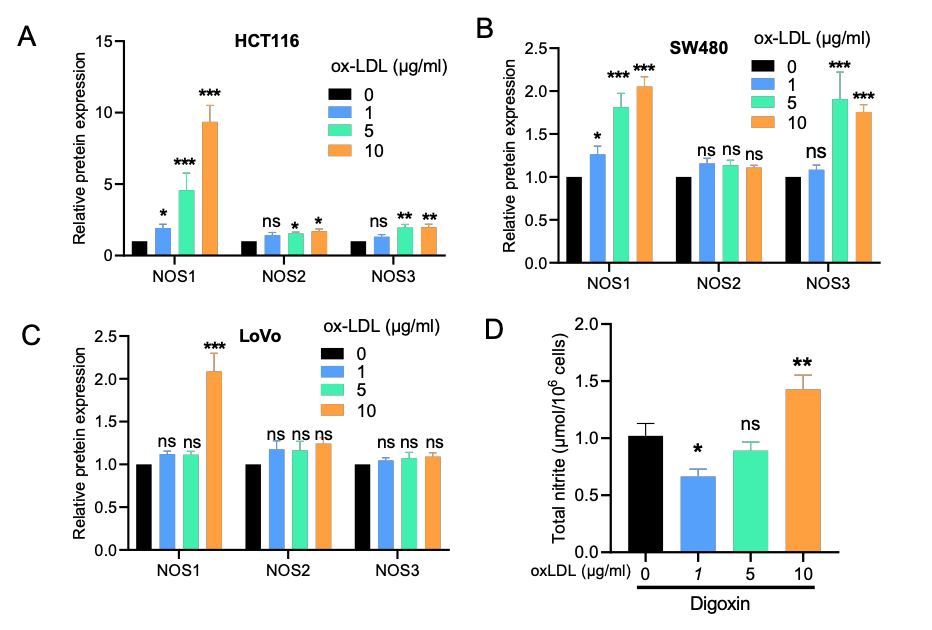
**

**Supplementary Fig. S5.** Densitometric quantification of NOSs protein expression in HCT116 **(A)**, SW480 **(B)**, and LoVo **(C)** cell lines treated with increasing doses of oxLDL for 24 h. Cells treated with DMSO were equated to 1. **(D)** Changes of total nitrite levels in oxLDL and Digoxin-treated HCT116 cells. Two-tailed Student’s t-test. Error bars represent ± SD. ns: not significant, *p < 0.05, **p < 0.01 ***p < 0.001, ns: not significant. Data represent mean ± SD of at least three independent experiments.

**Supplementary Fig. S6**

**
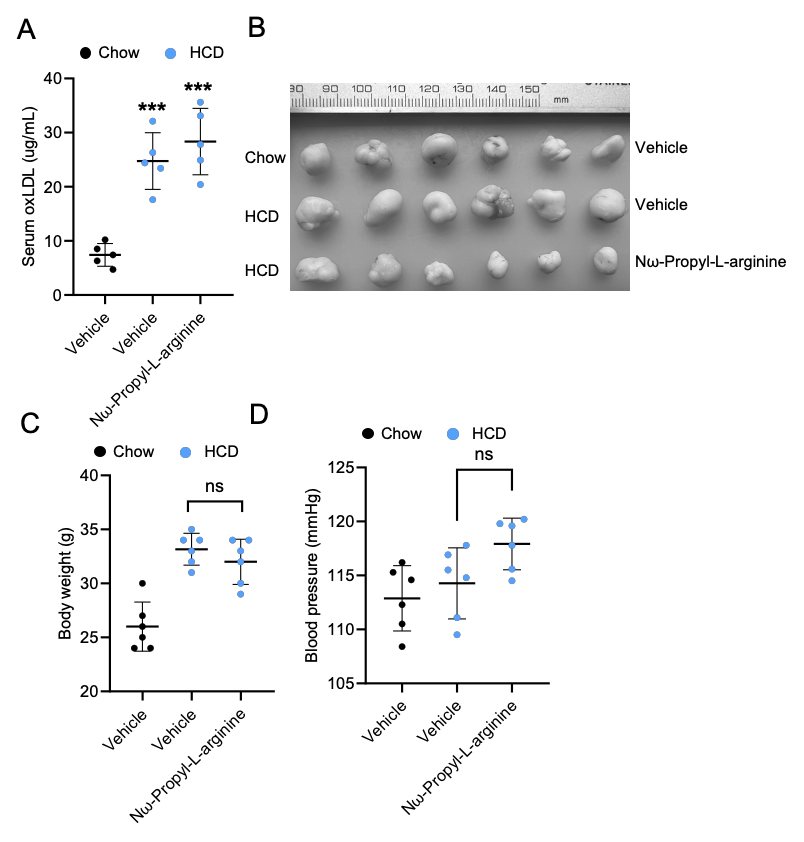
**

**Supplementary Fig. S6**. **Pharmacologic blockade of NOS1 is a promising therapeutic strategy for hypercholesterolemia-related CRC. (A)** The serum oxLDL levels in HCD-induced hypercholesterolemia nude mice. **(B)** Representative images of xenograft tumors of HCT116 cells formed in HCD-induced hypercholesterolemia nude mice treated with vehicle or Nω-Propyl-L-arginine (n = 6 mice per group). **(C)** Body weight of vehicle- or Nω-Propyl-L-arginine -treated mice at 4 weeks after the starting dose of drug administration. **(D)** Blood pressure of vehicle- or Nω-Propyl-L-arginine -treated mice at 4 weeks after the starting dose of drug administration. Two-tailed Student’s t-test. Error bars represent ± SD. ns: not significant, ****p* < 0.001. Data represent mean ± SD of at least three independent experiments.

**Supplemental Table S1. shRNA sequences are listed.**

| shLOX-1#1 | GCTCGGAAGCTGAATGAGAAA |
| --- | --- |
| shLOX-1#2 | CCTGGGATTAGTAGTGACCAT |
| shScr | TTCCTGGAACAATTGCTTTTA |

**Supplemental Table S2. Primers sequences are listed.**

| LOX-1 (Human) | Forward | TTGCCTGGGATTAGTAGTGACC |
| --- | --- | --- |
|  | Reverse | GCTTGCTCTTGTGTTAGGAGGT |
| NOS1 (Human) | Forward | TTCCCTCTCGCCAAAGAGTTT |
|  | Reverse | AAGTGCTAGTGGTGTCGATCT |
| NOS2 (Human) | Forward | TTCAGTATCACAACCTCAGCAAG |
|  | Reverse | TGGACCTGCAAGTTAAAATCC |
| NOS3 (Human) | Forward | TGATGGCGAAGCGAGTGAAG |
|  | Reverse | ACTCATCCATACACAGGACCC |
| HIF1A (Human) | Forward | GAACGTCGAAAAGAAAAGTCTCG |
|  | Reverse | CCTTATCAAGATGCGAACTCACA |
| GAPDH (Human) | Forward | GGAGCGAGATCCCTCCAAAAT |
|  | Reverse | GGCTGTTGTCATACTTCTCATGG |
| BNIP3 (Human) | Forward | CAGGGCTCCTGGGTAGAACT |
|  | Reverse | CTACTCCGTCCAGACTCATGC |
| CA9 (Human) | Forward | GGATCTACCTACTGTTGAGGCT |
|  | Reverse | CATAGCGCCAATGACTCTGGT |
| EGLN3 (Human) | Forward | CTGGGCAAATACTACGTCAAGG |
|  | Reverse | GACCATCACCGTTGGGGTT |
| SLC2A1 (Human) | Forward | GGCCAAGAGTGTGCTAAAGAA |
|  | Reverse | ACAGCGTTGATGCCAGACAG |
| VEGFA (Human) | Forward | AGGGCAGAATCATCACGAAGT |
|  | Reverse | AGGGTCTCGATTGGATGGCA |
| ChIP negative locus (Human) | Forward | TTTTCAAGGTCGGGAGTGATG |
|  | Reverse | ACTTTTTCATATGCCACCTCCTTT |
| ChIP NOS1-HRE (Human) | Forward | GCTTCCCTTTCCTCTTCTCTTG |
|  | Reverse | GATGGAGAAGGGGAAACATAGG |
